# Disulfidptosis modification patterns are involved in the immune microenvironment regulation of septic acute respiratory distress syndrome

**DOI:** 10.1590/1414-431X2025e14932

**Published:** 2026-03-09

**Authors:** Qian Zhang, Junke Ge

**Affiliations:** 1 Intensive Care Unit, Shandong Provincial Third Hospital, Shandong University, Shandong, Jinan, China

**Keywords:** Disulfidptosis, Septic ARDS, Immune microenvironment, Diagnostic model, Sepsis, Subtype

## Abstract

Disulfidptosis is a new form of programmed cell death. However, there is limited information available regarding the impact of disulfidptosis on septic acute respiratory distress syndrome (ARDS). The 16 disulfidptosis-related genes (DRGs) were collected from a previous study. Gene expression data of sepsis and septic ARDS samples were downloaded from the Gene Expression Omnibus database. The risk score model in septic ARDS was constructed based on the DRGs, followed by the investigation of immune microenvironment in septic ARDS patients. Furthermore, septic ARDS patients were divided into different subtypes based on disulfidptosis modification patterns, and their immune characteristics were investigated. Finally, the differentially expressed genes among different subtypes were identified, and a diagnostic model was constructed. The risk score model based on 6 DRGs was constructed to distinguish sepsis patients from septic ARDS patients, with good performance. The immune microenvironment in septic ARDS patients was slightly different from sepsis patients. Additionally, septic ARDS patients were divided into two subtypes based on DRGs. Finally, three diagnostic models based on 3 hub genes were constructed to classify the two subtypes in septic ARDS patients. Our findings indicated that disulfidptosis might play a role in the immune microenvironment of septic ARDS.

## Introduction

Acute respiratory distress syndrome (ARDS) is triggered by inflammatory lung injury and manifests clinically as acute hypoxic respiratory failure (1). It is characterized by enduring inflammation, proliferation of parenchymal cells, and aberrant collagen deposition (1). ARDS accounts for 10% of admissions to intensive care units, affecting over 3 million patients worldwide annually (2). Pneumonia, non-pulmonary sepsis, and aspiration of stomach contents are the most common risk factors for ARDS (3). Septic ARDS accounts for 32% of ARDS cases and is associated with greater disease severity and higher mortality rates (27-37%) (3,4). ARDS survivors often experience cognitive decline, depression, persistent skeletal muscle weakness, and other sequelae (5). Therefore, it is urgent to explore novel and potential diagnostic strategies for septic ARDS.

Disulfidptosis, a new form of programmed cell death, occurs in cancer cells with high-expression of SLC7A11 under glucose starvation conditions (6). The excessive aggregation of intracellular disulfide molecules results in disulfide stress (7). By providing the reducing power, nicotinamide‐adenine dinucleotide phosphate (NADPH) can inhibit cysteine inter- and intramolecular disulfide bonding (8). A lack of NADPH results in abnormal binding of disulfide bonding within actin cytoskeletal proteins, leading to the collapse of the actin network and cell death (9). Alterations in the actin cytoskeleton are important for endothelial cell inflammation, which is a central event in the pathogenesis of ARDS (10). However, the role of disulfidptosis in septic ARDS is unclear.

In this study, we used bioinformatics analysis to explore the role of disulfidptosis in septic ARDS patients. We first evaluated the expression levels of 16 disulfidptosis-related genes (DRGs) between sepsis and septic ARDS patients, followed by the construction of a risk score model and evaluation of immune cells. Furthermore, disulfidptosis-related subtypes were identified according to 16 DRGs expression patterns, and the level of immune cells between the subtypes was compared. Additionally, a diagnostic model was developed. Our findings may contribute to understanding the pathology of septic ARDS.

## Material and Methods

### Data collection

Two datasets (GSE66890 and GSE32707) were obtained after searching the Gene Expression Omnibus database. GSE66890 includes blood samples from 28 sepsis patients and 29 septic ARDS patients. GSE32707 includes blood samples from 34 non-septic ICU control subjects, 30 patients with sepsis alone on day 0, and 18 patients with septic ARDS on day 0. There was no sample overlap between the sepsis and septic ARDS datasets (PMID: 25795726; PMID: 22461369). A total of 16 DRGs were collected from the previous study, including *GYS1*, *NDUFS1*, *NDUFA11*, *NUBPL*, *LRPPRC*, *SLC7A11*, *SLC3A2*, *RPN1*, *NCKAP1*, *OXSM*, *CYFIP1*, *WASF2*, *ABI2*, *BRK1*, *RAC1*, and *SLC2A1* (7). All the analyses were performed using R software version 3.5.3.

### Characterization of DRGs in septic ARDS

The Rcircos package of R was used to visualize the distribution of DRGs on chromosomes. To investigate the relationship among the 16 DRGs, the protein-protein interaction (PPI) network was performed based on the STRING database (https://string-db.org/). The Spearman correlation was used to evaluate the correlations among the DRGs in all samples. The expression levels of 16 DRGs between the sepsis and septic ARDS samples were compared using the *t*-test, with a P-value <0.05.

To study the contribution of DRGs in the pathology of septic ARDS, the least absolute shrinkage and selection operator (LASSO) regression analysis was used to exclude the relatively unimportant DRGs. Then, the septic ARDS diagnostic model was constructed based on the remaining DRGs. Furthermore, the receiver operating curve (ROC) analysis, the area under the curve (AUC), and confusion matrix were performed to evaluate the accuracy of the diagnostic model.

### Immune microenvironment analysis

To evaluate the immune cell levels of the 23 types of immune cells and the immune response activity in the septic ARDS patients, the single-sample gene set enrichment analysis (ssGSEA) was performed using the Bioconductor package “GSVA”. The *t*-test was applied to compare the immune cell levels and immune response activity between the two groups. In addition, Spearman's correlation analysis was used to assess the correlation between the DRGs and differential immune cells, as well as the correlation between DRGs and differential immune responses. In addition, four hypoxia-related gene sets (Manalo hypoxia up, Mense hypoxia up, Hallmark hypoxia, and Harris hypoxia) from the MSigDB database (https://www.gsea-msigdb.org/gsea/msigdb/) were selected to assess the hypoxic status of septic ARDS with ssGSEA.

### Identification of disulfidptosis-based subtypes

The 29 blood samples from septic ARDS in GSE66890 and the 18 blood samples in GSE32707 were combined using the “combat” function in the “sva” package of R to remove the batch effect. Finally, a total of 47 septic ARDS samples were collected.

According to the expression levels of 16 DRGs, unsupervised cluster analysis was performed to divide septic ARDS patients into different groups with PAM algorithm and “Euclidean” distances in “ConsensusClusterPlus” package in R. Meanwhile, “ggplot2” package in R was used to profile the principal component analysis (PCA). The expression of DRGs, the level of immune cells, and specific immune response activity were compared among different subtypes using the *t*-test. The differentially expressed genes (DEGs) among different subtypes were obtained using the “limma” package (|log_2_ Fold Change| >1 and false discovery rate <0.05). The DEGs were visualized by volcano plot and heatmap.

### Weighted gene co-expression network analysis (WGCNA) and function enrichment analysis

The “WGCNA” package in R was applied to investigate the hub genes related to septic ARDS. The function “hclust” was used to cluster samples to detect outliers. Three outliers, GSM812705, GSM812721, and GSM812737, were removed to avoid affecting downstream analyses. Then, the function “pickSoftThreshold” was used to select suitable soft thresholding power to construct scale-free network. A power of β=7 as the soft threshold was used to construct the scale-free network. The degree was set as 0.90. Next, the adjacency matrix was transferred into the topological overlap matrix (TOM) and 1-TOM. Genes with similar expression patterns were clustered together and divided into modules with default parameters according to the “cutyreeDynamic” function. Since the modules identified by the dynamic tree cutting algorithm may be similar, they were merged at a height of 0.25. The gene significance (GS) and module membership (MM) were calculated to select candidate genes. Genes with |GS| >0.4 and |MM| >0.6 in the hub modules were selected as the subtype related genes. Then, these genes were intersected with DEGs to gain the hub genes in subtypes. The Gene Ontology (GO) and Kyoto Encyclopedia of Genes and Genomes (KEGG) enrichment analyses were performed to explore the function of hub genes using the David database (P-values <0.05).

### Feature selection and model construction

The machine learning algorithms were used to screen the biomarkers associated with subtypes. First, LASSO regression analysis was used to select the feature genes from hub genes with the R package “glmnet”. Next, the random forest (RF) algorithm was used to rank the feature genes in descending order. The optimal number of genes was obtained by adding one gene at a time using the RF algorithm and 10-fold cross-validation via top-down approach. Finally, RF, support vector machine (SVM), and decision tree (DT) classification models were constructed using “randomForest”, “e1071”, and “rpart” packages. A 10-fold cross-validation was applied to prevent overfitting, and it was also used to formulate predictive models. Subsequently, the diagnostic power of three models was evaluated based on ROC curves.

### Real time qPCR (RT-qPCR)

A total of 20 blood samples collected from 7 patients with sepsis and 13 patients with septic ARDS were selected to perform RT-qPCR. Everyone provided written informed consent. The total RNA was extracted using HiPure Liquid RNA Mini Kit (Magen, China), and the FastQuant cDNA First Strand Synthesis Kit (Tiangen, China) was used for reverse transcription. RT-qPCR was performed using SuperReal PreMix Plus (SYBR Green, Tiagen). The primer sequences are reported in Supplementary Table S1. This study was approved by the Ethics Committee of Shandong Provincial Third Hospital. All procedures adhere to the tenets of the Declaration of Helsinki, and all patients signed informed consent.

## Results

### Characterization of DRGs in septic ARDS

**Figure 1:**
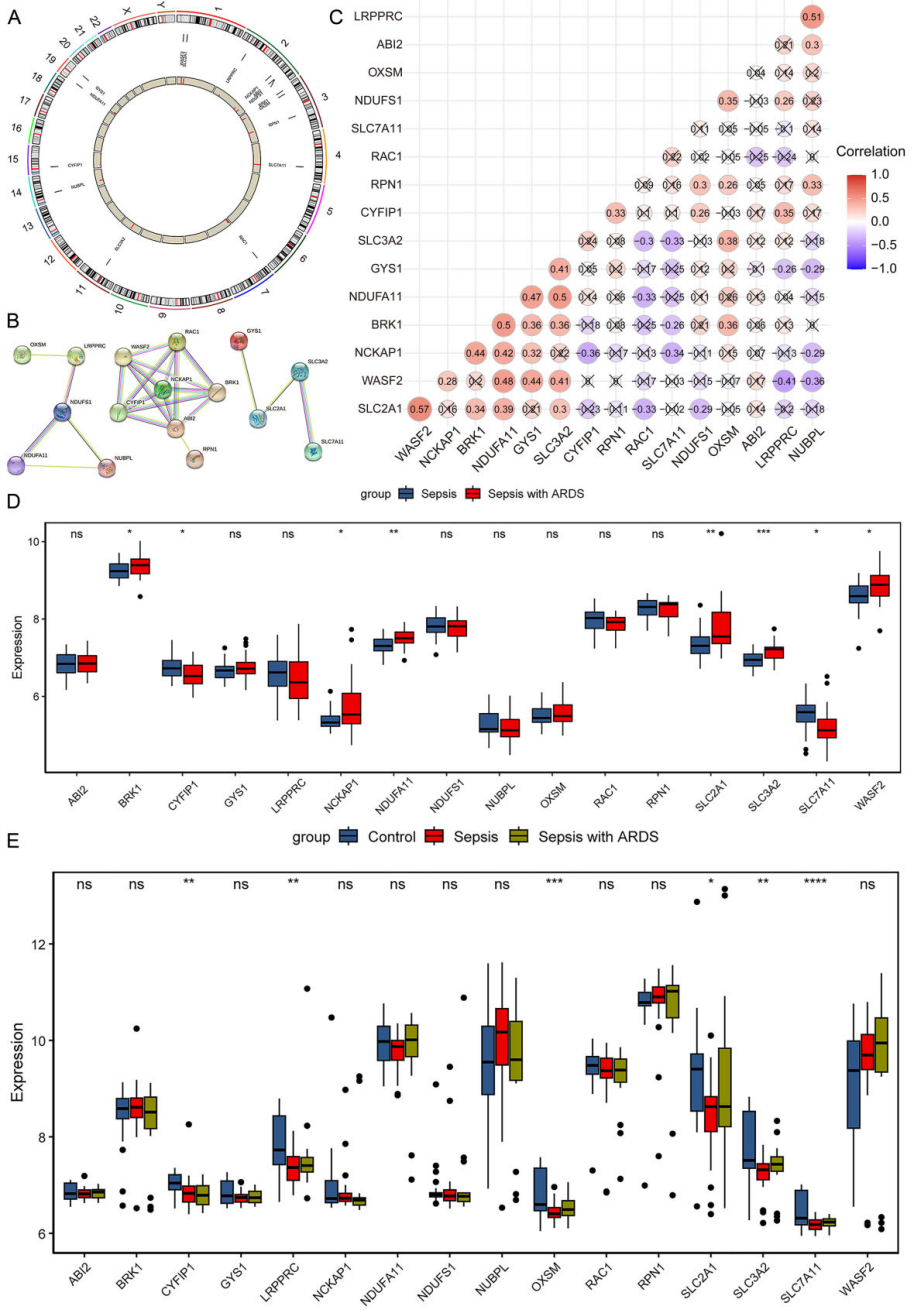
Expression landscape of 16 disulfidptosis-related genes (DRGs) in septic acute respiratory distress syndrome (ARDS) in the GSE66890 dataset. **A**, Distribution of the 16 DRGs on the chromosomes. **B**, Protein-protein interaction (PPI) network of the 16 DRGs. **C**, Correlation analysis of the 16 DRGs. **D**, Box plot showing the expression of the 16 DRGs in the GSE66890 dataset. **E**, Box plot showing the expression of the 16 DRGs in the GSE32707 dataset. Data are reported as median and interquartile range. *P<0.05, **P<0.01, ***P<0.001, ****P<0.0001, ns: not significant (Student's *t*-test in D; ANOVA in E).

The distribution of the 16 DRGs on the chromosomes is shown in Figure 1A, indicating that DRGs were found in chromosome regions 1, 2, etc. As expected, DRGs were not found in the sex chromosome regions X or Y. The interaction relationships of these DRGs are illustrated in Figure 1B, with most DRGs having a close connection with others. Next, the correlation analysis among the 16 DRGs was performed, exhibiting the highest positive correlation between *SLC2A1* and *WASF2* at 0.57, and the highest negative correlation between *LRPPRC* and *WASF2* at -0.41 in all samples (Figure 1C). The expression levels of the 16 DRGs in the control, sepsis, and septic ARDS samples were further investigated. Among the 16 DRGs, 8 showed differential expression between sepsis and septic ARDS samples and 6 showed differential expression among control, sepsis, and septic ARDS samples (P<0.05, Figure 1D and E).

**Figure 2:**
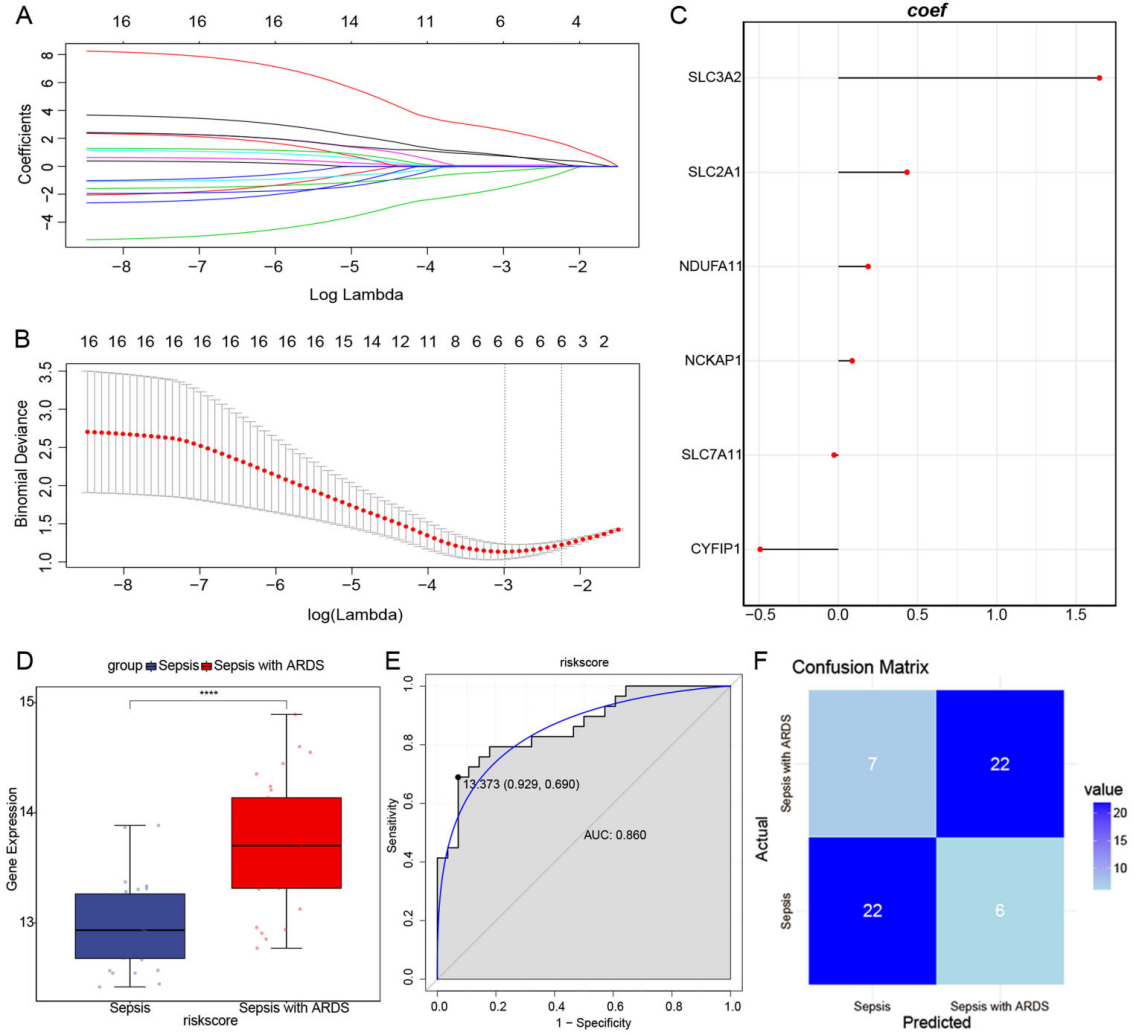
Disulfidptosis-related genes (DRG)-related risk score model distinguished septic acute respiratory distress syndrome (ARDS) patients from sepsis patients. **A**, LASSO regression coefficients of the 16 DRGs. **B**, Ten-fold cross-validation for selecting the lambda. **C**, Lollipop chart showing the coefficient of the six genes in the risk score model. **D**, Risk score between sepsis and septic ARDS. Data are reported as median and interquartile range. **E**, ROC analysis of the risk score model in GSE66890. **F**, Confusion matrix for the risk score model. ****P<0.0001 (Student's *t*-test).

The LASSO regression analysis was conducted to further explore the 16 DRGs' contribution to the pathogenesis of septic ARDS. LASSO regression analysis was performed to reduce the number of genes (Figure 2A and B). Finally, six genes were selected to construct a risk model (Figure 2C): Risk score = *CYFIP1* × (-0.49296064) + *NCKAP1* × 0.08669857 + *NDUFA11* × 0.18760621 + *SLC2A1* × 0.43265271 + *SLC3A2* × 1.64689253 + *SLC7A11* × (-0.02702564). The risk score model can distinguish sepsis patients from septic ARDS patients well (Figure 2D). The AUC value of the risk score model was 0.860 (Figure 2E), which was higher than the AUC value of a single gene (Supplementary Figure S1). The confusion matrix for the risk score model is shown in Figure 2F. The numbers of true positive (TP, the number of patients that are accurately diagnosed as septic ARDS), true negative (TN, the number of patients that are accurately diagnosed as sepsis), false positive (FP, the number of patients that are misdiagnosed as septic ARDS), and false negative (FN, the number of patients that are misdiagnosed as sepsis) were 22, 22, 6, and 7, respectively. The above results indicated that disulfidptosis may play a role in septic ARDS.

### DRGs and septic ARDS

**Figure 3:**
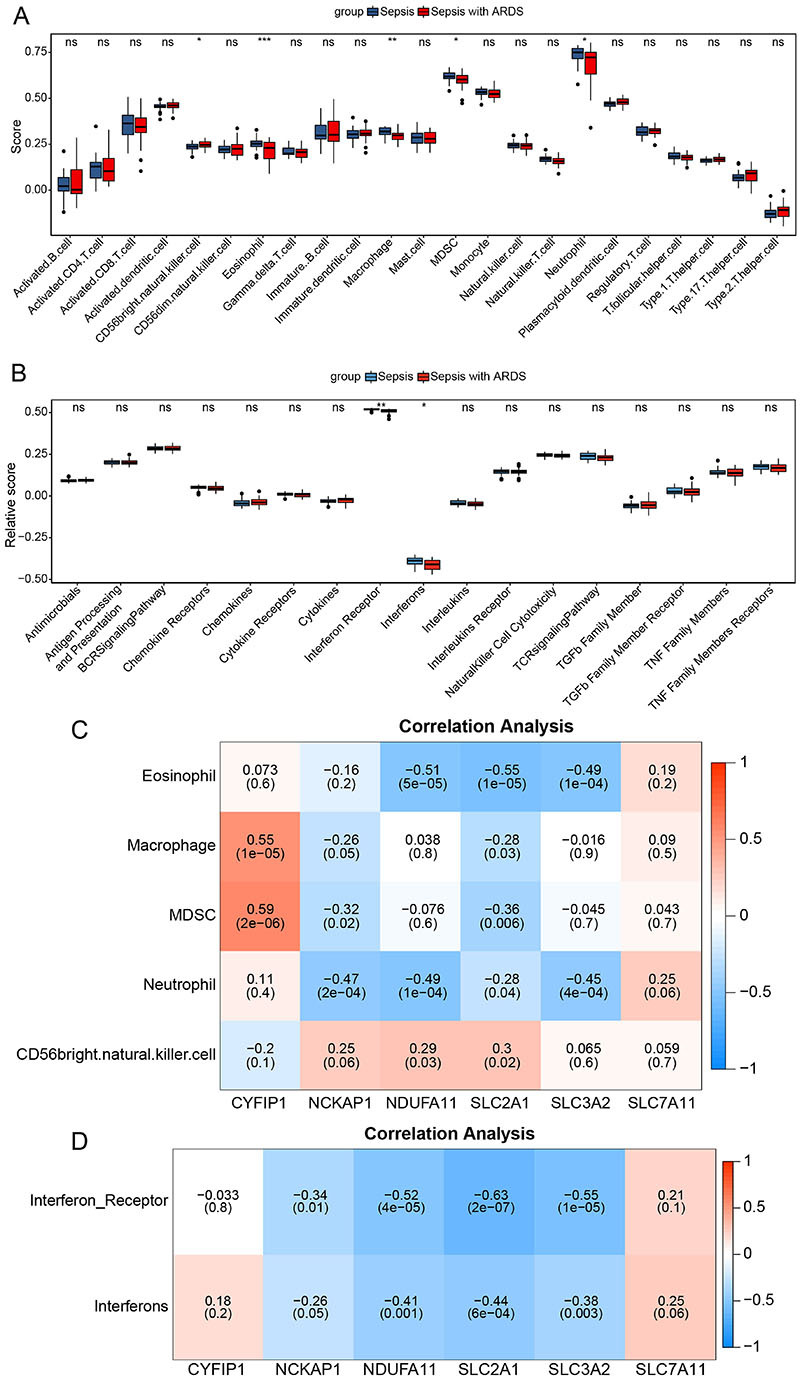
Immune microenvironment characteristics between sepsis and septic acute respiratory distress syndrome (ARDS) patients. **A**, Abundance of immune infiltrating cells between sepsis and septic ARDS samples. **B**, Activity of immune reaction between sepsis and septic ARDS. Data are reported as median and interquartile range. **C**, Heatmap of the correlation between immune infiltrating cells and six disulfidptosis-related genes (DRGs). **D**, Heatmap of the correlation between immune response activity and six DRGs. *P<0.05, **P<0.01, ***P<0.001, ns: not significant (Student's *t*-test).

The immune microenvironment in septic ARDS patients was slightly different from sepsis patients. The levels of five immune cells were significantly changed in septic ARDS, including elevated levels of CD56 bright natural killer cells, and levels of eosinophils, macrophages, myeloid-derived suppressor cells (MDSC), and neutrophils were decreased (Figure 3A). In addition, the activity of immune reaction (interferons and interferon receptors) also showed differences between the two groups (Figure 3B). Correlation analysis further indicated that the expression of six DRGs was associated with five immune cells. MDSC exhibited a positive correlation with *CYFIP1* (r=0.59), while the eosinophil level was negatively correlated with *SLC2A1* (r=-0.55, Figure 3C). Moreover, interferon and interferon receptors were negatively correlated with *NCKAP1*, *NDUFA11*, *SLC2A1*, and *SLC3A2* (Figure 3D). Additionally, we found that among four hypoxia-related gene sets, the Hallmark Hypoxia and Harris Hypoxia gene sets were activated in septic ARDS (FDR <0.05; Supplementary Figure S2).

### Disulfidptosis modification patterns in septic ARDS

**Figure 4:**
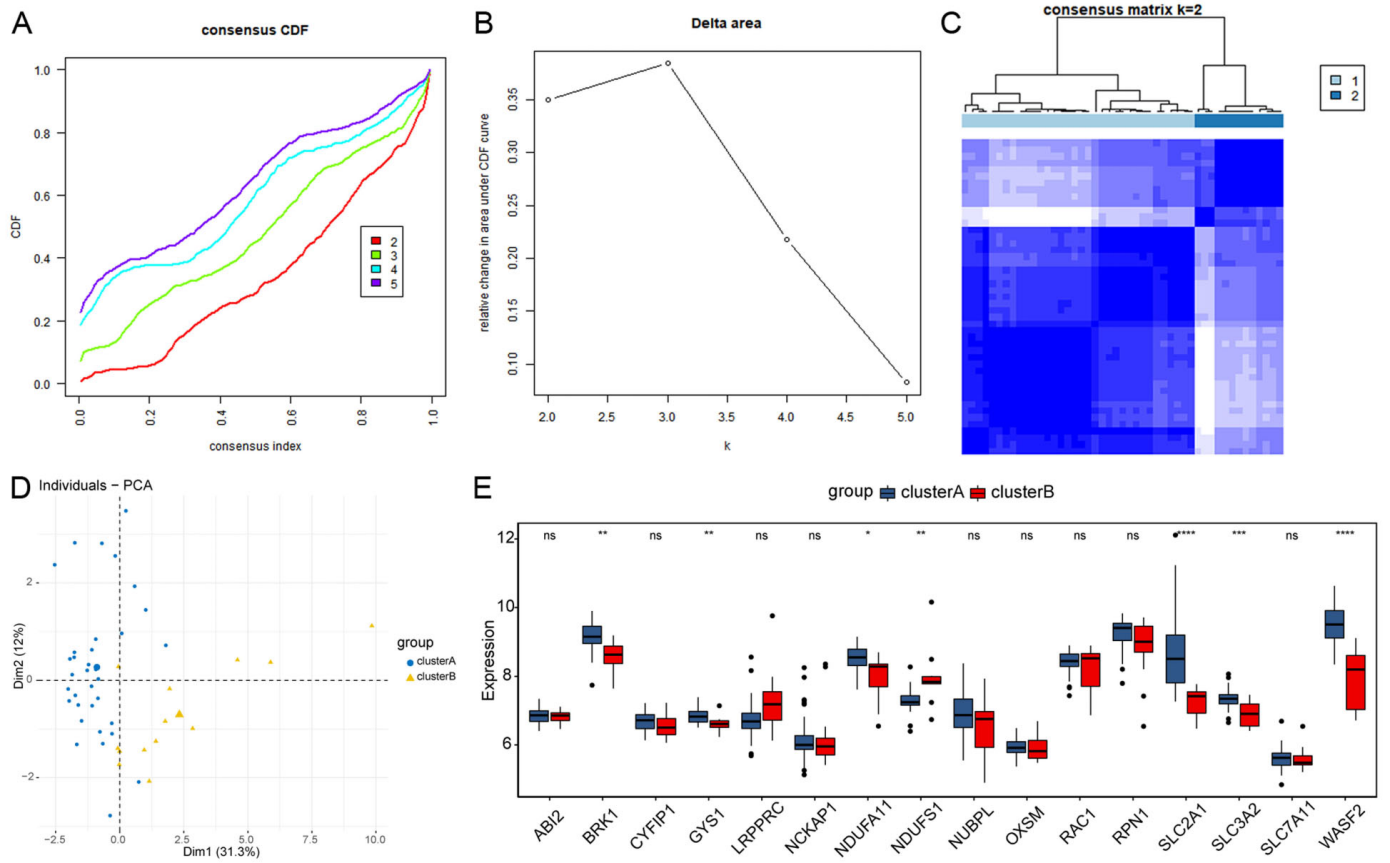
Identification of disulfidptosis modification patterns in septic acute respiratory distress syndrome (ARDS) patients. **A**, Cumulative distribution function (CDF) curves with k=2-5. **B**, Relative change in the area under the CDF curves for k=2-5. **C**, Matrix heatmap of septic ARDS subtypes. **D**, Principal component analysis (PCA) for subtypes. **E**, Expression of 16 disulfidptosis-related genes (DRGs) between cluster A and cluster B. Data are reported as median and interquartile range. *P<0.05, **P<0.01, ***P<0.001, ****P<0.0001, ns: not significant (Student's *t*-test).

To investigate disulfidptosis modification patterns in septic ARDS, unsupervised consensus clustering analysis for septic ARDS samples was performed based on the expression of 16 DRGs. Consensus clustering revealed that k=2 was identified with optimal clustering stability (Figure 4A and B). Then, the septic ARDS patients were divided into two subtypes (cluster A and cluster B), and the PCA showed a significant interval between the two subtypes (Figure 4C and D). The expression levels of 16 DRGs between two subtypes are shown in Figure 4E.

### Immune microenvironment characteristics

**Figure 5:**
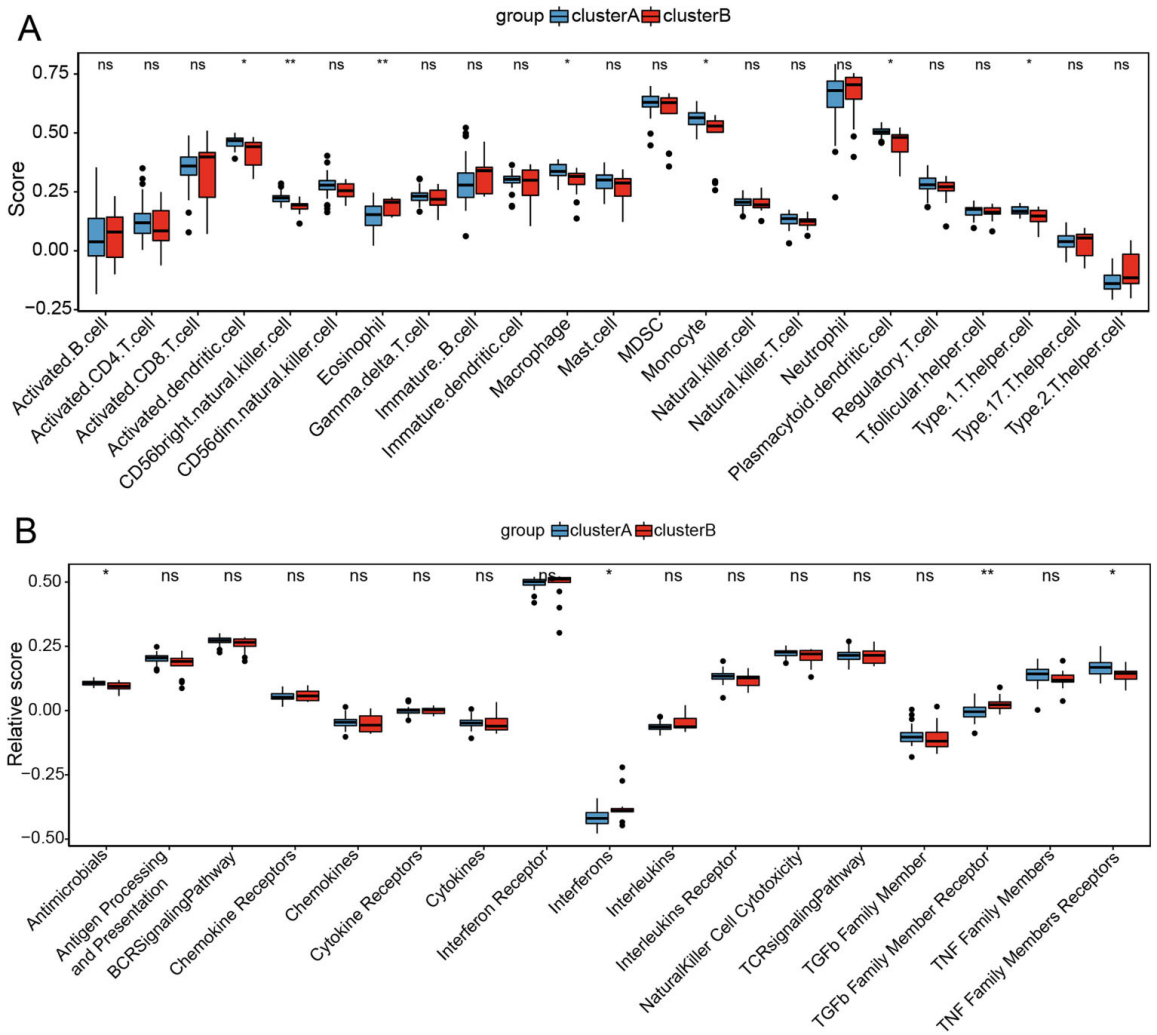
Immune microenvironment characteristics in different disulfidptosis-based subtypes. **A**, Difference of immune cell level between cluster A and cluster B. **B**, Difference in immune response activity between cluster A and cluster B. Data are reported as median and interquartile range. *P<0.05, **P<0.01, ns: not significant (Student's *t*-test).

Immune microenvironment analysis indicated that cluster A had a higher level of activated dendritic cells, CD56 bright natural killer cells, macrophages, monocytes, plasmacytoid dendritic cells, and type 1 T helper cells, while eosinophils were enriched in cluster B (P<0.05, Figure 5A). Antimicrobials and tumor necrosis factor family member receptors were more activated in cluster A than in cluster B (P<0.05, Figure 5B). Compared with cluster A, interferons and transforming growth factor beta family member receptors were more activated in cluster B (P<0.05). In addition, we further evaluated the hypoxic state of the two subtypes and found that the four hypoxic-related gene sets were significantly enriched in cluster A (FDR <0.05, Supplementary Figure S3).

### Identification of the hub genes

**Figure 6:**
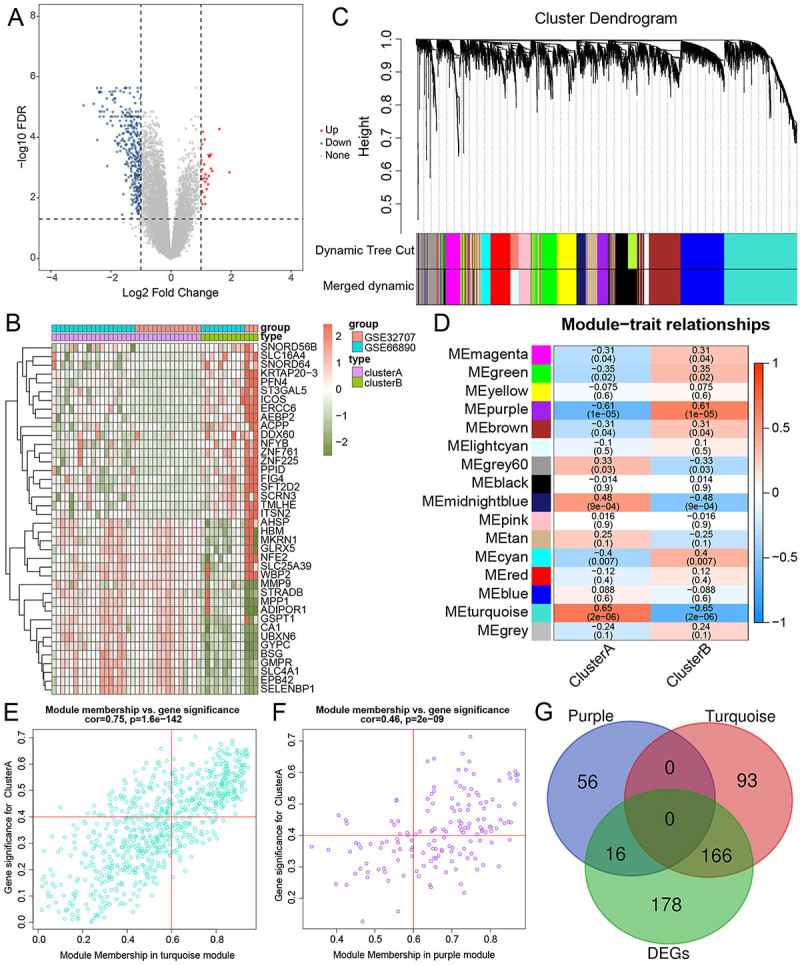
Identification of hub genes in septic acute respiratory distress syndrome (ARDS) samples. **A**, Volcano plot of the differentially expressed genes (DEGs) between cluster A and cluster B. **B**, Heatmap of the top 20 up/down-regulated DEGs. **C**, Gene dendrogram (top) was obtained, and primary modules (middle) and merged modules (bottom) are shown by dynamic tree cut. **D**, Relationship of module eigengenes and subtypes. Scatterplot of genes in the turquoise (**E**) and purple (**F**) modules. **G**, Hub genes shown in the Venn diagram.

The differential expression analysis revealed a total of 360 DEGs in cluster B compared with cluster A, including 29 up- and 331 down-regulated genes (Figure 6A). The heatmap of the top 20 up/down-regulated genes is displayed in Figure 6B. Then, WGCNA revealed sixteen gene modules, each exhibiting a distinct modification pattern corresponding to its associated gene expression (Figure 6C). The correlation analysis between features (cluster A and cluster B) and network modules revealed the highest positive correlation between cluster A and the turquoise module (r=0.65) and between cluster B and the purple module (r=0.61, Figure 6D). According to |MM| >0.6 and |GS| >0.4 parameters, a total of 331 genes related to subtypes were found, of which 259 were in turquoise, and 72 were in the purple modules (Figure 6E and F). Subsequently, by overlapping these 331 genes with 360 DEGs in two subtypes, 16 genes were detected after intersecting with the purple module, and 166 genes were detected following the intersection with the turquoise module, resulting in a total of 182 hub genes (Figure 6G).

### Function enrichment analysis

**Figure 7:**
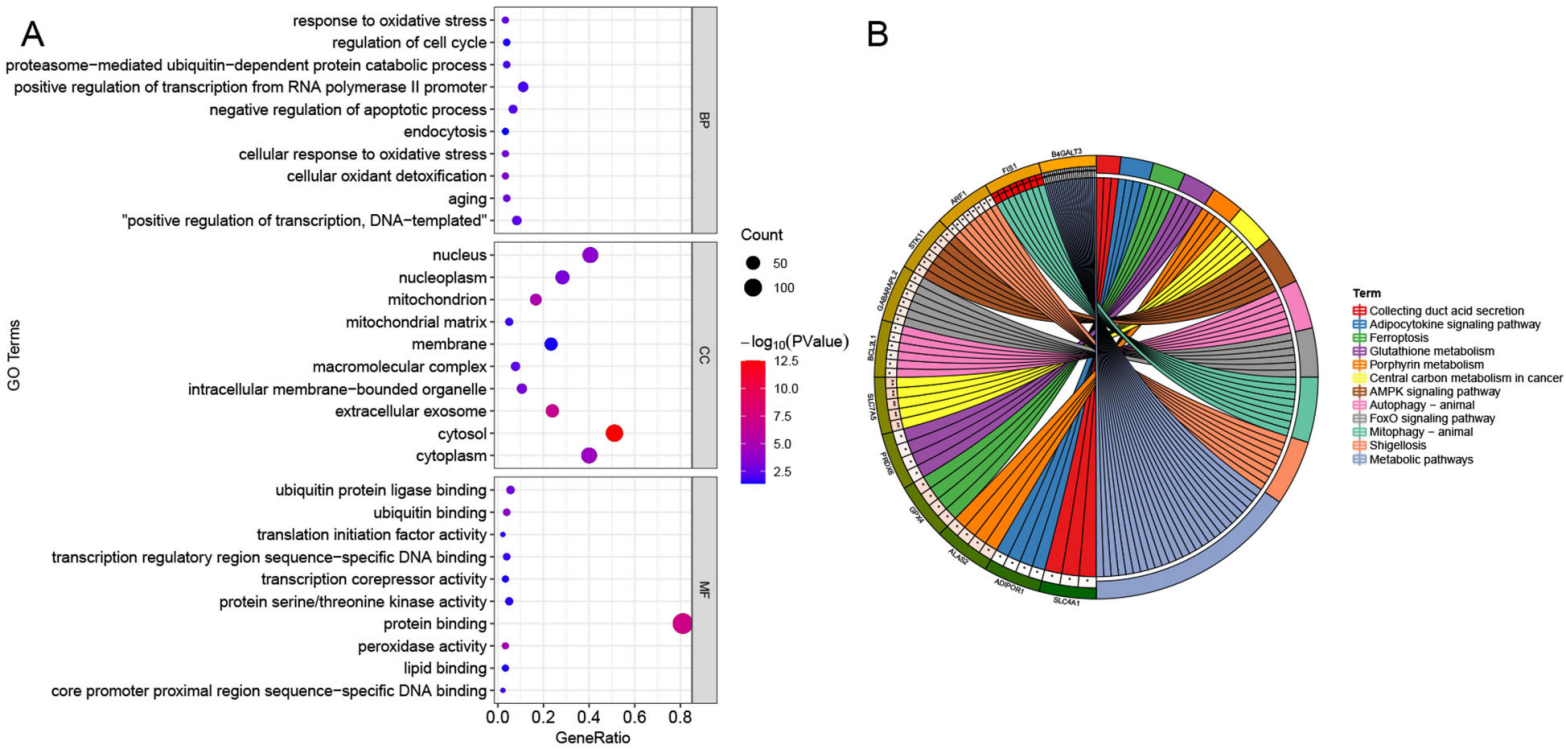
Function enrichment analysis. Gene Ontology (GO; **A**) and Kyoto Encyclopedia of Genes and Genomes (KEGG; **B**) pathways enrichment analyses for hub genes in septic acute respiratory distress syndrome (ARDS).

The GO enrichment analysis showed that the 182 hub genes were mainly involved in oxidative stress, protein catabolic process, and cell cycle-related pathways (Figure 7A). Based on the KEGG analysis, they were mainly involved in the regulation of energy metabolism, such as adipocytokine signaling pathway, ferroptosis, glutathione metabolism, porphyrin metabolism, central carbon metabolism in cancer, AMPK signaling pathway, FoxO signaling pathway, and mitophagy (Figure 7B).

### Diagnostic model construction for subtypes

**Figure 8:**
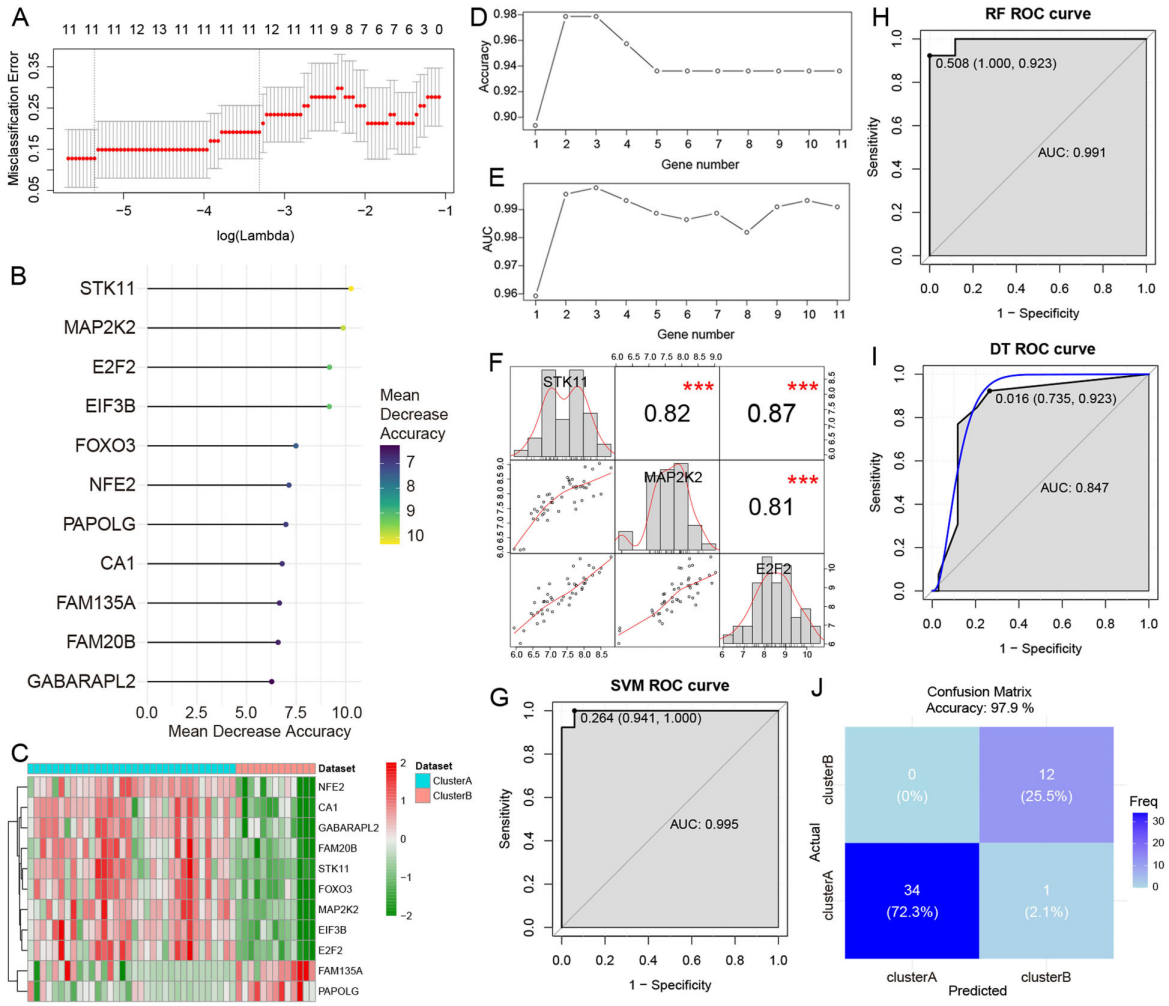
Diagnostic model construction for subtypes. **A**, LASSO regression analysis identified the optimal lambda. **B**, Lollipop chart showing the mean decrease accuracy of 11 genes. **C**, Heatmap showing the expression of 11 hub genes in the two subtypes. **D** and **E**, Results of the 10-fold cross-validation showed that the highest accuracy was achieved when the number of genes was 3. **F**, Correlation analysis among *STK11*, *MAP2K2*, and *E2F2*. **G**-**I**, ROC analysis of the support vector machine (SVM; **G**), random forest (RF; **H**), and decision tree (DT; **I**) models; **J**, Confusion matrix for the SVM model.

A total of 11 genes were screened after LASSO regression analysis of 182 hub genes (Figure 8A). Then, these genes were ranked based on their mean descending precision (Figure 8B). The heatmap of these 11 genes is shown in Figure 8C. The 10-fold cross-validation showed that the highest accuracy was achieved when the number of genes was three (*STK11*, *MAP2K2*, and *E2F2*) (Figure 8D and E). Correlation analysis revealed strong correlations among the three genes (Figure 8F). Subsequently, the three genes were used in the classification model using RF, SVM, and DT algorithms. The SVM model exhibited an AUC of 0.995, demonstrating a perfect sensitivity of 100% and a remarkable specificity of 94.1% (Figure 8G). In addition, the RF model achieved an AUC of 0.991, with a sensitivity of 92.3% and a perfect specificity of 100% (Figure 8H). However, the DT model showed a comparatively lower AUC of 0.847, with a sensitivity of 92.3% and a specificity of 73.5% (Figure 8I). The confusion matrix for the SVM model is shown in Figure 8J, with TP, TN, FP, and FN of 12, 34, 1, and 0. In addition, except the DT model, the SVM and RF models achieved higher AUC values than a single gene (Supplementary Figure S4).

### RT-qPCR validation

**Figure 9:**
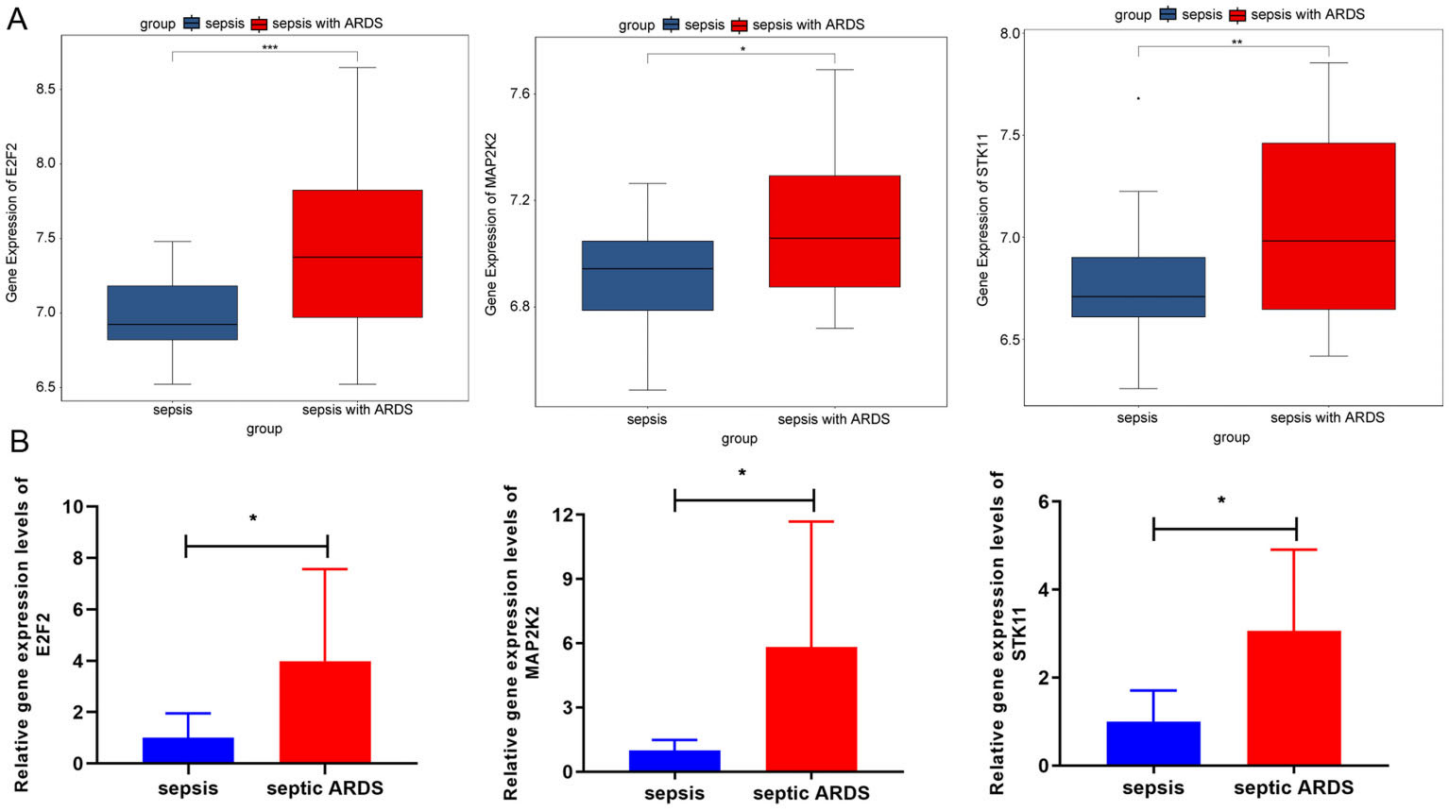
The expression levels of *STK11*, *MAP2K2*, and *E2F2* between sepsis and septic acute respiratory distress syndrome (ARDS) patients in GSE66890 (**A**) and clinical samples (**B**). *P<0.05, **P<0.01, ***P<0.001 (Student's *t*-test).

To validate our exploratory bioinformatics analysis, RT-qPCR analysis was performed. The expression levels of *STK11*, *MAP2K2*, and *E2F2* were up-regulated in the septic ARDS patients compared with sepsis patients, consistent with the results in the GSE66890 dataset (Figure 9).

## Discussion

ARDS is a severe inflammatory lung disease that causes damage to the lung tissue, fluid-filled alveoli, and immune infiltration, ultimately leading to breathlessness and hypoxemia (9,11). In sepsis, actin regulates endothelial barrier function and vascular permeability (12). Disulfidptosis is a new form of cell death, which mainly affects the actin cytoskeleton (6). However, it is not clear whether disulfidptosis plays a role in septic ARDS. For the first time, we divided septic ARDS patients into two subtypes based on the expression levels of DRGs, and constructed the diagnostic models based on 3 hub genes that distinguish the two subtypes effectively.

In this study, 8 of 16 DRGs were differentially expressed between sepsis and septic ARDS groups, including decreased *CYFIP1* and *SLC7A11*, and increased *BRK1*, *NCKAP1*, *NDUFA11*, *SLC2A1*, *SLC3A2*, and *WASF2*. CYFIP1, NCKAP1, and WASF2 are the core subunits of the WASp-family verprolin homologous protein (WAVE) complex. BRK1, also known as HSPC300, is also one of the components of the WAVE complex and interacts with other subunits to regulate the function of the complex (13). The WAVE complex plays a key role in regulating the actin cytoskeleton, influencing various cell processes, such as cell movement, morphology, signal transduction, apoptosis, cytokinesis, endocytosis, and migration (14,15). The WAVE complex transmits the signal to the Arp2/3 complex through RAC1, a small GTPase that interacts with CYFIP1, and the activation of the Arp2/3 complex triggers actin polymerization in disulfidptosis (16,17). The pathological hallmark of sepsis and ARDS is the impairment of the endothelial barrier, and the actin skeleton is closely related to the endothelial cell barrier (18). Actin dynamics play a crucial role in the regulation of endothelial barrier functions and neutrophil recruitment during sepsis (12). NDUFA11 is one of the subunits of the membrane-bound mitochondrial respiratory chain complex I, and the reduction of NDUFA11 leads to the reduction in levels of ATP and the increase of NADH (19). The interaction between ATP and the purinergic receptor P2X7 can activate macrophages, inducing the production of inflammatory mediators, including cytokines (e.g., IL-1β) as well as nitrogen and oxygen-reactive species, thereby triggering inflammation (20). During sepsis, purinergic signaling regulates the migration of core inflammatory cells and the production of inflammatory mediators (21).

SLC7A11 (also known as xCT), solute carrier family 7 members 11, and SLC3A2 (also known as CD98hc), solute carrier family 3 members 2, are the two subunits that make up the xCT complex. The xCT complex is a heterodimer of proteins responsible for cystine transport, exporting intracellular glutamate and importing extracellular cystine in a 1:1 ratio (22). SLC3A2 acts as a chaperone for a single transmembrane protein, maintaining the stability and proper membrane localization of the SLC7A11 protein. SLC7A11 serves as the catalytic subunit within the xCT complex, responsible for importing cystine that subsequently undergoes reduction to form cysteine (23). SLC3A2 was initially recognized as a surface antigen characteristic of activated lymphocytes, and it regulates inflammation by activating IL-18 in natural killer cells. *SLC2A1* encodes a crucial glucose transporter within the mammalian blood-brain barrier, facilitating energy-independent glucose transport into the brain (24). Lei et al. (25) reported that the expression of *SLC2A1* may be a risk factor for sepsis-associated delirium. Exosomes from sepsis patients regulate heart cell apoptosis and glycolysis via the hsa-miR-1262/*SLC2A1* signaling pathway (26). All the above results suggested that the disulfidptosis may participate in the regulation of septic ARDS.

The immune microenvironment in septic ARDS patients was slightly different from sepsis patients, including differences in the level of immune cells and the activity of immune reaction, interferons, and interferon receptors. Eosinophils are type II inflammation effector cells that may be crucial in ARDS development and progression (27). About 40 years ago, studies had reported that the number of eosinophils in patients with ARDS decreased (28,29). Additionally, eosinophils alleviate neutrophilic inflammation and are protective against ARDS (27). Macrophages play pro-inflammatory and anti-inflammatory roles in different pathological stages of sepsis or ARDS, depending on the immune microenvironment (30,31). In the early stage of ARDS, M1-like macrophages release inflammatory mediators, heightening the inflammatory response. In the late stage of ARDS, these macrophages transition to an M2-like state, producing anti-inflammatory factors, which can contribute to immune dysfunction and organ damage (31). MDSCs are immature bone marrow cells with immunosuppressive and inflammatory characteristics (32). The excessive proliferation of MDSCs caused by immunosuppression significantly contributes to secondary infections and mortality in late-stage sepsis (33). Mo et al. (34) found that the number of MDSCs was significantly increased in patients with septic ARDS by performing single-cell RNA-seq analysis of peripheral blood. In mouse models of sepsis, there was a decrease in the phagocytic capacity of inflammatory cells and a reduction in the expression of cytokines and chemokine receptors (35). In severe sepsis, interferon-β can reverse these effects (35). In our study, the levels of eosinophils, macrophages, and MDSCs were decreased in septic ARDS samples. Macrophages and MDSC were positively correlated with the *CYFIP1*. Eosinophils were negatively correlated with *NDUFA11*, *SLC2A1*, and *SLC3A2*. Interferons and interferon receptors were negatively correlated with *NDUFA11*, *SLC2A1*, and *SLC3A2*. These findings may imply the potential role of the four DRGs in immune regulation of septic ARDS.

Biomarkers associated with ARDS can help us understand disease pathogenesis and its progression, contributing to identifying ARDS subgroups and facilitating treatment. In this study, three diagnostic models including three genes (*STK11*, *MAP2K2*, and *E2F2*) were constructed, and we found that the expression levels of these genes were all significantly higher in the septic ARDS group than in the sepsis group. *STK11*, the serine-threonine kinase 11 gene, is involved in cellular energy metabolism. Under low ATP levels, *STK11* phosphorylates and activates AMP-activated protein kinase (AMPK) (36). AMPK activation leads to the phosphorylation and inactivation of enzymes that are crucial for macromolecular synthesis while promoting catabolism to preserve cellular energy homeostasis (36). Grégoire et al. (37) reported that restoring AMPK activity with metformin could reduce lung inflammation and limit progression of alveolar injury and pulmonary fibrosis in ARDS patients. MAP2K2 (MAPK kinase 2) belongs to the MAP kinase family and participates in intracellular signaling networks. Gong et al. (38) reported that a MAP2K2 variant is associated with mortality of patients with ARDS. In a clinical mouse model of ARDS (acute lung injury), MAP2K2 promotes and maintains selective pro-inflammatory pathway activation, and leukocyte MAP2K2 is a key regulator of acute lung injury duration (38). *E2F2* belongs to the *E2F* gene family, which plays a crucial role in regulating cell proliferation, differentiation, and apoptosis. *E2F2* serves as a negative regulator of the immune response by impeding the proliferation of activated lymphocytes in silencing *E2F2* mice (39). Thus, all the above results implied that these three genes might participate in the development of septic ARDS.

Some limitations remained in this study. The sample sizes for RT-qPCR validation were small. Hence, large-scale sample validation and *in vivo* and *in vitro* experiments are necessary. *In vitro* studies using ARDS models to verify the role of the identified DRGs in disulfidptosis and immune regulation, and *in vivo* animal models of septic ARDS to investigate the roles of the identified genes and pathways in disease progression will be included in our next work plan. We also plan to collect additional patient samples during subsequent clinical investigations to further validate the reliability of our findings. Additionally, the pathology of septic ARDS is complex, and we only collected data at the mRNA level. It is urgent to clarify the role of disulfidptosis in septic ARDS from multiple perspectives. These further investigations will enhance the scientific and clinical significance of our findings.

## Conclusion

In summary, our study is the first to systematically analyze the relationship between disulfidptosis and the immune microenvironment in septic ARDS based on published datasets. Our findings highlighted the crucial role of disulfidptosis in the immune microenvironment of septic ARDS, which may contribute to understanding the underlying mechanism of septic ARDS.

## Data Availability

The datasets used and/or analyzed during the current study are available from the corresponding author on reasonable request.

## Supplementary Materials

Supplementary Material Click to view [pdf].
