# Supplementary material for: Disulfidptosis modification patterns are involved in the immune microenvironment regulation of septic acute respiratory distress syndrome
Source: Braz J Med Biol Res. 2026 Mar 9;59:e14932. doi: 10.1590/1414-431X2025e14932 (PMC12971013; doi:10.1590/1414-431X2025e14932)
Supplement: Supplementary Material [file 1414-431X-bjmbr-59-e14932-suppl.pdf]

Figure S1. ROC analysis of *CYFIP1*, *NCKAP1*, *NDUFA11*, *SLC2A1*, *SLC3A2*, *SLC7A11*.

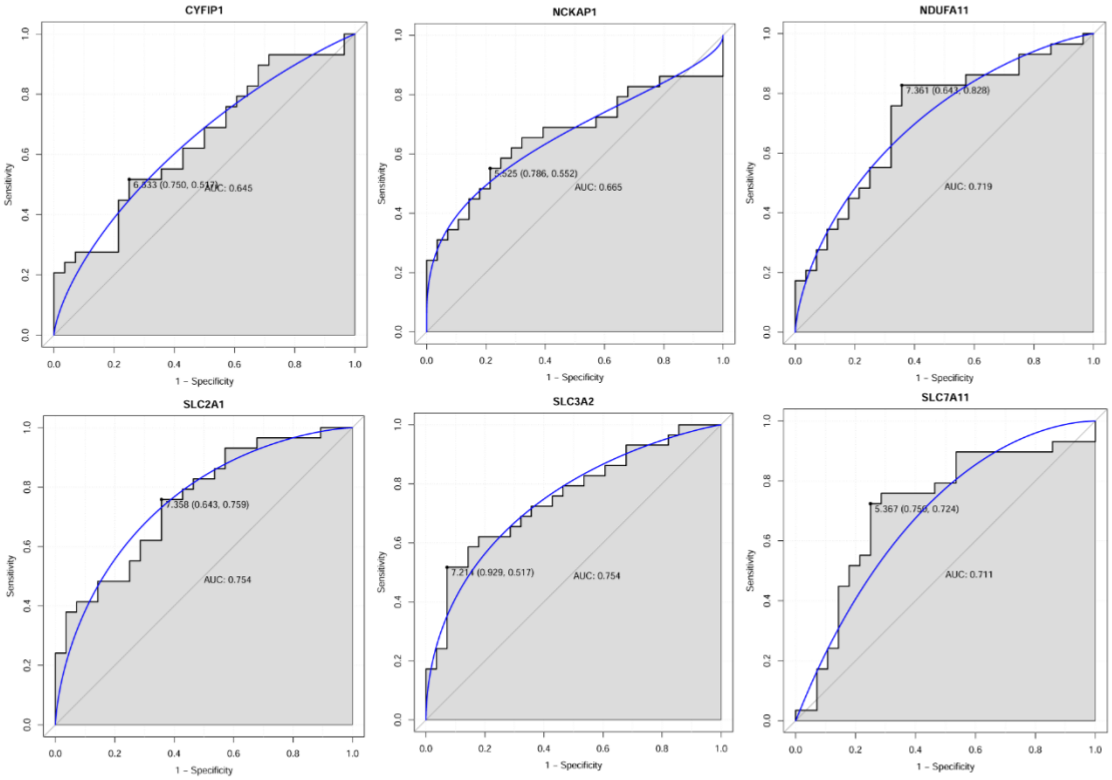

Figure S2. ssGSEA analysis showing enrichment of the hypoxia-related gene sets in septic acute respiratory distress syndrome (ARDS) patients.

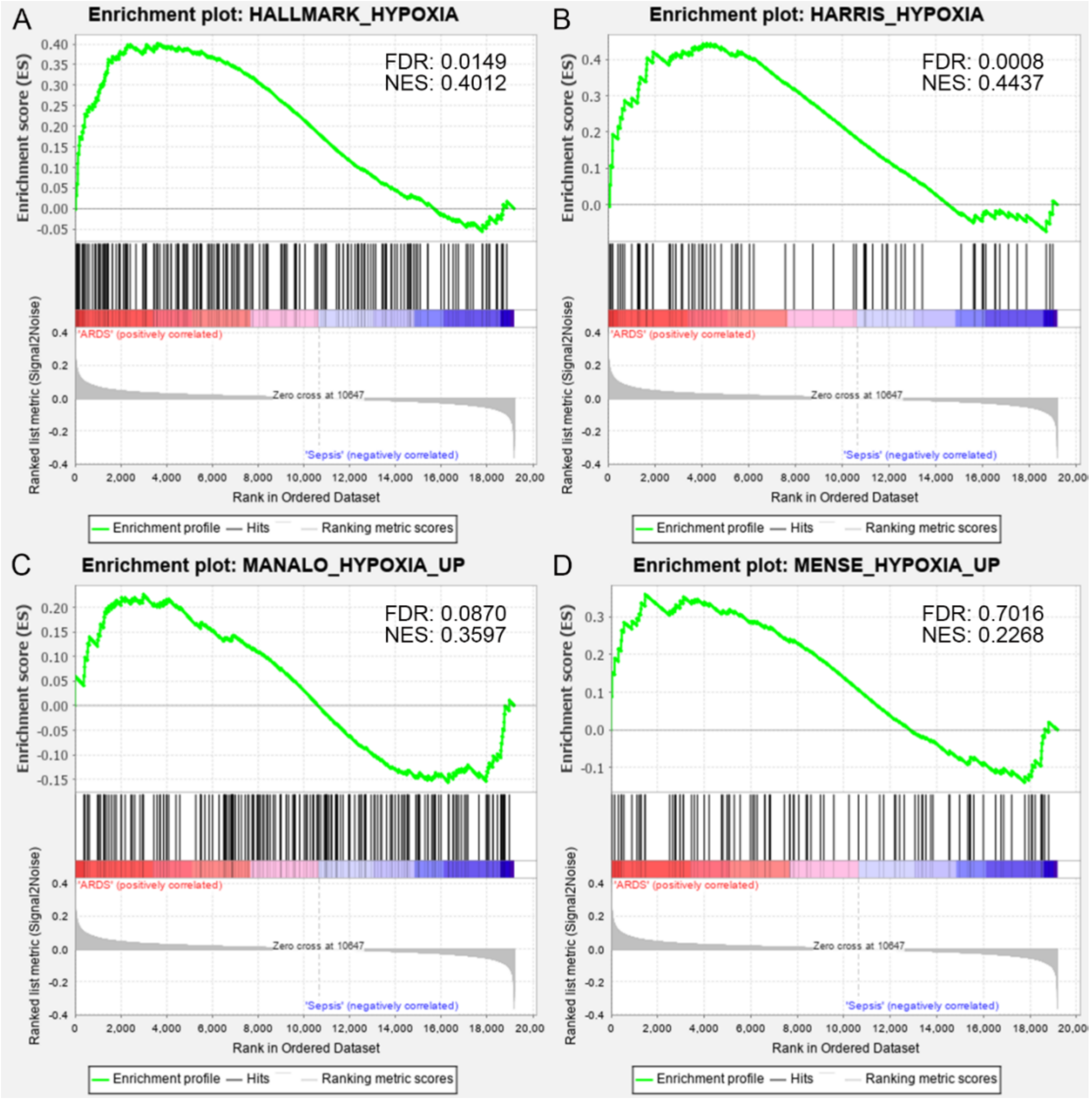

Figure S3. ssGSEA analysis shows enrichment of the hypoxia-related gene sets in different subtypes.

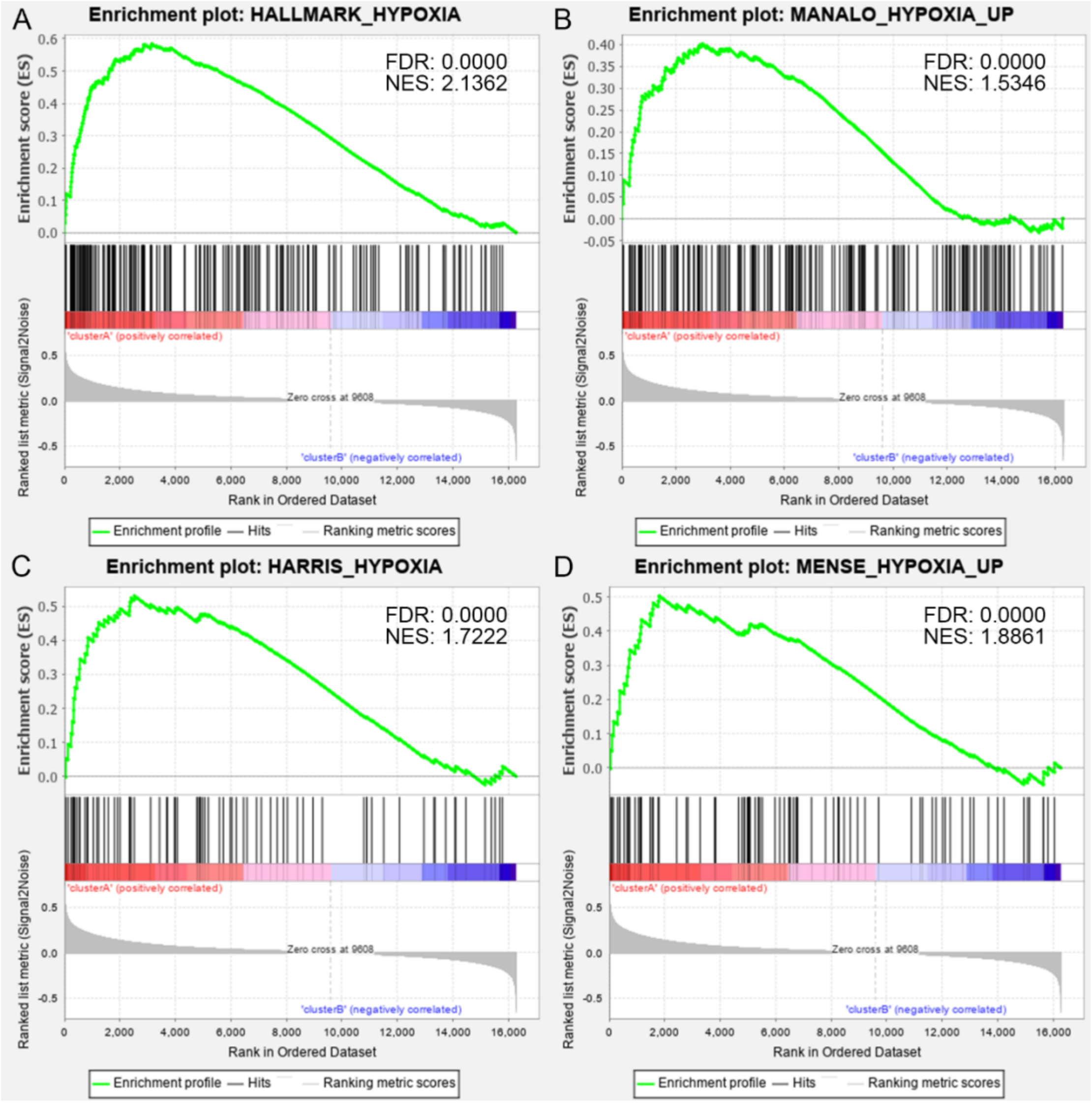

Figure S4. ROC analysis of *E2F2*, *MAP2K2*, and *STK11*.

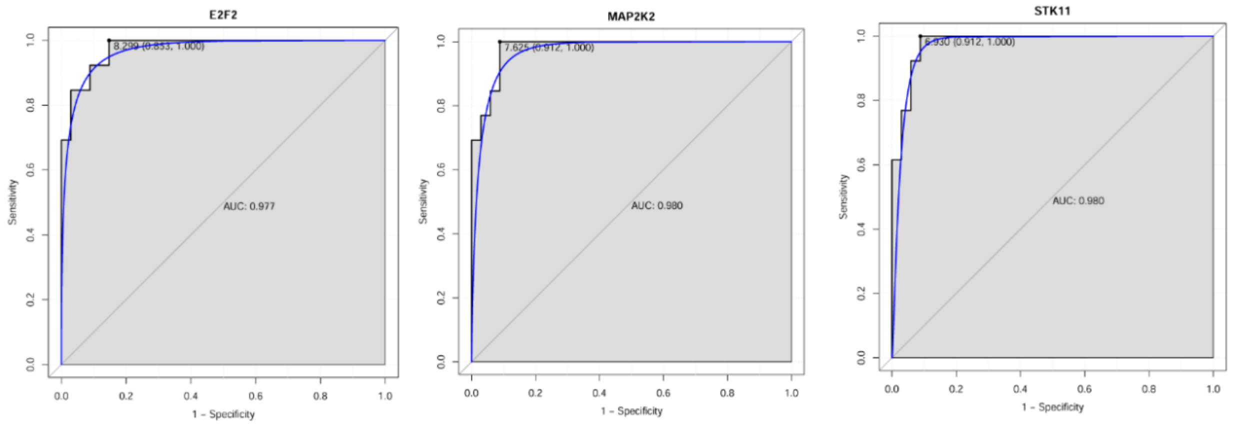

Table S1. Primer sequences.

Name	Species	Forward primer	Reverse primer
GAPDH	Human	GGAGCGAGATCCCTCCAAAAT	GGCTGTTGTCATACTTCTCATGG
ACTB	Human	CATGTACGTTGCTATCCAGGC	CTCCTTAATGTCACGCACGAT
STK11	Human	AGGGATGCTTGAGTACGAACC	GTCCTCCAAGTACGGCACC
MAP2K2	Human	GCTTCTACGGGGCCTTCTAC	TGGTGCTTCTCTCGGAGGTA
E2F2	Human	CGTCCCTGAGTTCCCAACC	GCGAAGTGTCATACCGAGTCTT
